# Supplementary material for: A State‐Resolved Single‐Cell and Spatial Atlas Reveals IDH‐Associated Differences in Malignant and Immune Architecture in Diffuse Glioma
Source: MedComm (2020). 2026 Oct 4;7(10):e71022. doi: 10.1002/mco2.71022 (PMC13634174; doi:10.1002/mco2.71022)
Supplement: Supplementary file 1 [file MCO2-7-e71022-s001.docx]

**A State-Resolved Single-Cell and Spatial Atlas Reveals** *IDH***-Associated Differences in Malignant and Immune Architecture in Diffuse Glioma**

Ashraf Zaman¹^,^²^,#^, Emily Holton¹^,#^, Madhavi Pandya¹, Genevieve Shillington^1,3^, Cecile Escher¹, Kavitha Krishna Sudhakar¹, Agota Tuzesi^1^, Ashish Mehta^1^, Eric Lam¹, Rachael Zekanovic¹, Michael Sughrue^4^, Charlie Teo^5^, Jacky Yeung^6^, Walter Muskovic¹, Joseph E. Powell¹^,3,7,*^

1. Translational Genomics Program, Garvan Institute of Medical Research, Sydney, Australia

2. School of Clinical Medicine, Faculty of Medicine and Health, University of New South Wales (UNSW), Sydney, Australia

3. School of Biomedical Sciences, Faculty of Medicine and Health, UNSW Sydney, Australia

4. Omniscient Neurotechnology, Sydney, Australia

5. Cingulum Health Clinic, Sydney, Australia

6. Yale School of Medicine, New Haven, USA

7. UNSW Cellular Genomics Futures Institute, University of New South Wales, Sydney, Australia

\# Equal contribution

**Correspondence** *Prof. Joseph E. Powell <j.powell@unsw.edu.au >

**Keywords:** diffuse glioma; single-cell RNA sequencing; spatial transcriptomics; transcriptional heterogeneity; astrocyte-like progenitor cells (ALPC)

# **Abstract**

Adult-type diffuse gliomas are defined by the isocitrate-dehydrogenase (*IDH*) axis, yet how this biology constrains malignant cell-state repertoires and their immune-modulatory potential remain unclear. We profiled 69 tumors using single-cell RNA sequencing (scRNA-seq), single-nucleus RNA sequencing (snRNA-seq), and spatial transcriptomics, and resolved seven recurrent malignant cell states: astrocyte-like progenitor cells (ALPC), hypoxia, cycling G1/S, cycling G2/M, oligo-lineage, neuro-oligo-lineage, and neuro-lineage. The *IDH* axis strongly biased state occupancy and malignant–immune composition, with *IDH*-mutant tumors dominated by differentiated lineage-like states and reduced immune infiltration, whereas glioblastoma (GBM) showed expansion of ALPC and hypoxia states alongside increased myeloid and lymphoid infiltration. Orthogonal analyses supported that ALPC-associated markers are expressed *in situ* and in patient-derived stem cell-permissive cultures, consistent with a stem-like, differentiation-competent malignant phenotype. State-resolved pathway analysis identified mitochondrial oxidative phosphorylation programs enriched in GBM ALPC and extracellular matrix remodeling, and chemotaxis-associated programs enriched in GBM hypoxia. Composition-controlled, patient-level interaction modeling identified a greater malignant-myeloid signaling interface in GBM, driven by SPP1–CD44/integrin signaling and confirmed by differential expression; GBM networks also featured TGFB and NECTIN modules, whereas *IDH*-mutant networks were dominated by APP/APOE–TREM2/TYROBP and MIF modules. These data suggest that the *IDH* axis shapes glioma behavior by constraining malignant state repertoires and biasing the deployment of state-specific metabolic and immunomodulatory programs.

# **Introduction**

# Adult-type diffuse gliomas comprise a molecularly defined spectrum of infiltrative brain tumors that remain difficult to cure and account for the majority of malignant primary central nervous system (CNS) tumors in adults [1]. The current World Health Organization (WHO) classification separates *IDH*-mutant astrocytoma and oligodendroglioma from *IDH*-wildtype glioblastoma (GBM), which differ in clinical course and therapeutic response [1]. For GBM, median survival remains limited, reflecting persistent biological barriers to durable control [2]. A major contributor to treatment failure is the extensive heterogeneity of diffuse gliomas, encompassing both tumor-intrinsic transcriptional plasticity and variability in immune infiltration and activation [3,4].Early work showed that GBM comprises diverse malignant programs linked to proliferation, hypoxia, and lineage-like differentiation [5], later formalized into a model in which cells transition among a constrained set of transcriptional states shaped by plasticity and genetic context [6]. Parallel studies in *IDH*-mutant gliomas identified lineage-linked hierarchies and demonstrated more differentiated oligodendroglial and neuronal features than in GBM [7,8]. Together, these studies support the idea that gliomas may share a limited set of malignant cell states, with downstream effects on aggressiveness and therapeutic vulnerability [6–8]. However, existing resources have often been subtype-focused, modality-limited, or underpowered for systematic, cross-entity comparisons, leaving open how a shared state repertoire is deployed across the adult-type diffuse glioma spectrum and how this relates to immune composition in patient tumors.

# The immune context in gliomas is particularly relevant due to differences in immune infiltration and immunotherapy responsiveness, yet the basis of these cellular differences remains unresolved [4,9]. Experimental and translational evidence suggests that mutant *IDH* activity and its oncometabolite-driven effects can dampen immune recruitment and activation, including suppression of STAT1-linked chemokine programs and reduced T-cell accumulation in model systems [10]. Large-scale profiling of recurrent disease further shows that immune phenotypes and malignant programs can co-evolve under therapeutic pressure, motivating analyses that link malignant states to immune composition rather than treating these as independent [9]. A key gap, therefore, is whether specific malignant cell states, particularly stem/peri-vascular-like and stress-adapted populations, carry distinct immune-modulatory potential, and whether GBM versus *IDH*-mutant tumors differ not only in the abundance of such states but also in the state-resolved pathways and inferred signaling interfaces through which malignant cells may engage myeloid and lymphoid compartments.

# Spatial transcriptomics enables us to localize genomic programs and immune niches within tissue architecture, which is valuable in glioma, given regional hypoxia, vascular specialization and variable immune infiltration [11,12].

# Here, we present a single-cell and spatial transcriptomic atlas of 69 adult-type diffuse gliomas spanning *IDH*-wildtype GBM and *IDH*-mutant astrocytoma and oligodendroglioma. Building on a shared state framework [13], we resolve seven recurrent malignant cell states and show that the *IDH* axis is associated with reproducible differences in state occupancy and malignant–immune composition. We focus on two GBM-enriched states, astrocyte-like progenitor cells (ALPC) and hypoxia-responsive malignant cells, and integrate orthogonal evidence supporting ALPC marker expression in patient material and stem cell-permissive phenotypes in patient-derived cultures. We further use state-resolved pathway analyses to identify functional specialization within GBM ALPC and hypoxia states and apply composition-controlled cell-cell interaction modeling to scRNA-seq data to infer state-associated malignant–immune communication modules. Finally, spatial transcriptomics provides tissue-scale context for the regional organization of malignant, glial, immune, and vascular-associated signals.

# **Results**

## Pan-diffuse glioma single-cell atlas

We generated a single-cell transcriptomic atlas from 69 adult-type diffuse glioma samples, comprising 44 glioblastomas (GBMs), 17 astrocytomas (ASTR), and 8 oligodendrogliomas (ODG) (**Figure 1A**). Diagnoses and molecular classifications were confirmed by histopathology and molecular markers. Among GBMs, 16 were primary (pGBM), and 28 were recurrent (rGBM); these were further stratified by O6-methylguanine-DNA methyltransferase (*MGMT*) promoter methylation status (14 methylated, 16 unmethylated). Detailed metadata for clinical and data generation are provided in **Table S1**.

Single-cell RNA-seq (scRNA-seq) data were generated for 44 fresh tumor specimens, yielding 563,798 high-quality cells, and single-nucleus RNA-seq (snRNA-seq) data were generated for 22 frozen specimens, yielding 75,132 high-quality nuclei. Additionally, spatial transcriptomics (Visium) was performed on 10 GBM samples (7 tumors profiled by both sc/snRNA-seq and spatial, and 3 by spatial only). Quality control metrics confirmed high transcriptomic complexity per cell (**Figure S1A**). Final cell/nucleus counts per sample are summarized in **Figure S1B**. Using canonical lineage markers, we annotated 15 distinct cell types (**Figures 1B and C**), and we confirmed that malignant cells were present by their inferred copy-number variation (CNV) profiles.

We next examined the cellular composition across *IDH*-mutant and *IDH*-wildtype (GBM) tumors (**Figure 1D**). *IDH*-mutant gliomas exhibited significantly lower proportions of non-tumor (microenvironmental) cells, with an average of 35.9 % cells compared to 57.5% (*p*=8.7x10^-3^) in *IDH*-wildtype counterparts. Histologically, *IDH*-mutant tumors often have well-defined boundaries with less infiltration into normal brain [14].Consistent with this, our data show that *IDH*-mutant gliomas have markedly reduced immune cell infiltration at the tumor margins. Conversely, GBMs had a higher fraction of infiltrating myeloid and T cells, indicating a more heterogeneous and immunologically active tumor microenvironment (TME). This observation aligns with the well-established invasive nature of GBM, [15].

Comparisons between the two sequencing modalities (fresh scRNA-seq vs. frozen snRNA-seq) revealed expected biases in immune cell recovery (**Figure S1C and Table S2**). In our data, myeloid cells accounted for ~27% of cells in scRNA-seq but only ~10% in snRNA-seq samples, consistent with reports that cryopreservation and nuclear isolation under-recover immune cells. T cells were also less represented in snRNA-seq (**Table S3**). [16].

## Malignant cell state heterogeneity across diffuse gliomas

Malignant cells constituted over half of all cells captured (56.1% in scRNA-seq and 47.7% in snRNA-seq). Building upon our previous work [13], we classified malignant cells into seven transcriptionally distinct states: astrocyte-like progenitor cells (ALPCs), hypoxia, G1/S cell-cycle (proliferative), G2/M proliferative, oligodendroglial (“oligo”), neuronal–oligodendroglial hybrid (“neuro-oligo”), and neuronal (“neuro”). Each tumor contains different proportions of malignant cell states (**Figure 2A**) and the states exhibit a unique gene expression signature (**Figure 2B**), collectively capturing the heterogeneity of glioma cells. Notably, the ALPC state showed high expression of astrocytic lineage markers (*GFAP*, *AQP4*, *MLC1*) as well as genes associated with stem-like properties in gliomas (*ITGB4*, *S100A4*), suggesting a dual identity of differentiated astrocytic features and stem-like characteristics.

To assess whether ALPCs exhibit stem-like properties in patient-derived material, we performed protein-level and *in vitro* functional assays. Immunofluorescence confirmed expression of ALPC-associated markers in tumor tissue (**Figure S2A**). Patient-derived glioma cultures maintained under serum-free stem cell conditions expressed ALPC markers, along with canonical glioma stem cell markers (*OLIG2*, *SOX2*), and formed neurospheres, consistent with self-renewal capacity (**Figures S2B, S3A**). BMP4 treatment induced a clear shift towards astrocytic differentiation, characterized by reduced expression of the stem-associated marker *OLIG2* and a corresponding increase in *GFAP* across four independent cultures (**Figure S3B, C**). The relative stability of *SOX2* and S100B likely signifies an intermediate transition toward an astrocytic fate rather than terminal differentiation, a finding consistent with previous studies [17, 18].We use the term 'stem-like' to describe a transcriptional and phenotypic state characterized by sphere-forming capacity, expression of stem-associated markers, and differentiation competence, consistent with standard usage in the field [19].*In vivo* functional studies will be required to address this directly.

Comparisons across glioma subtypes revealed significant shifts in the distribution of malignant states (**Figures 2A, C-E**). GBMs showed higher relative abundance of ALPC, hypoxia, and G2/M proliferative states (**Figure 2C**), mirroring the known clinical aggressiveness of GBM. Elevated ALPC fractions in GBM are notable, as ALPCs resemble glioma stem cell programs previously associated with treatment resistance and disease recurrence [20]. The hypoxic state in GBMs was characterized by upregulation of angiogenic and hypoxia-survival genes (*VEGFA*, *VIM*, *CAV1*, *CD9*, *ADM*), consistent with established hypoxic adaptation pathways [21]. G2/M-state cells expressed high levels of cell-cycle regulators (*CENPA*, *CCNB1*, *CCNB2*), reflecting the high proliferative index of GBMs [22].

In contrast, *IDH*-mutant astrocytomas and oligodendrogliomas were dominated by the differentiated “oligo”, “neuro-oligo”, and “neuro” states. These lineage-restricted states (oligodendrocyte-like and neuronal-like programs) align with prior observations that *IDH*-mutant gliomas follow a more differentiated, less invasive trajectory [23, 9]. For example, the oligo state expressed canonical oligodendrocyte markers (*OLIG1*, *OLIG2*), while the neuro-oligo state showed a hybrid expression of oligodendroglial (*OLIG2*) and neuronal differentiation (*SOX6*) markers, indicative of partial differentiation.Its predominance in *IDH*-mutant tumors is consistent with the more lineage-restricted, differentiated character of these gliomas compared to GBM.Accordingly, survival analysis in our cohort confirmed that patients with *IDH*-mutant gliomas (enriched for differentiated oligodendroglial/neuronal states) have significantly (*p*<1.0x10^-4^) better outcomes than patients with GBM (**Figure S4**).

We observed limited differences in the composition of the malignant state between primary and recurrent GBMs (**Figure 2D**). Recurrent tumors showed a trend toward higher ALPC fractions than primary tumors, but this increase was not statistically significant. Meanwhile, in *IDH*-mutant astrocytomas, we observed that grade 4 tumors contained higher fractions of proliferative G1/S and G2/M cells than lower-grade (grade 2–3) *IDH*-mutant astrocytomas (**Figure 2E**). This corresponds with their more aggressive clinical behavior and is consistent with research linking cell-cycle activation to malignant progression [24]. To identify the sources of the substantial inter-sample variability in malignant-state composition (**Figure 2B**), we decomposed compositional variance across all samples (PERMANOVA) and tested clinical and molecular covariates within each subtype (per-sample compositional regression, limma). Tumor type was a significant contributor to overall variance (R² = 0.16, P < 10⁻⁵). Within astrocytomas, grade 4 tumors had significantly higher Cycling G1/S proportions (t = 6.12, false discovery rate [FDR] = 1.8 × 10⁻⁴) and lower Neuro and Oligo proportions (t = −4.05, FDR = 2.7 × 10⁻³) than grade 2. No significant associations were detected within GBM or oligodendroglioma between state composition and sex, age, *MGMT* methylation status, tumor margin, or primary/recurrent status.

## T and Natural Killer (NK) cell landscapes in adult-type diffuse glioma

We next analyzed the T and NK cell compartments using scRNA-seq data, as snRNA-seq samples yielded very limited recovery of T and NK cells (<50 nuclei per sample and essentially no NK cells), precluding robust cohort-wide lymphocyte quantification from snRNA-seq. Clustering of 16,234 single T and NK cells (across tumors) identified seven subpopulations based on canonical markers: CD4⁺ T cells, CD8⁺ T cells, activated regulatory T cells (Tregs), proliferative (cycling) lymphocytes, interferon-stimulated (ISG-high) T cells, CD56^bright^ NK cells, and CD56^dim^ NK cells (**Figures 3A and B**).

The relative lymphocyte abundance captured by scRNA-seq was higher in GBMs than in *IDH*-mutant tumors **(Figure 3C and D)**. Moreover, recurrent GBMs showed a higher median T-cell fraction than primary GBMs, with substantial inter-tumor variability (**Figure 3C**), suggesting an immunological impact of therapy or tumor evolution.These findings show immune microenvironmental divergence between *IDH*-wildtype and *IDH*-mutant gliomas [25] and provide evidence that treatment (and tumor recurrence) can induce increased immune cell recruitment in GBM [26].

Transcriptional profiling revealed functional differences between T cells infiltrating GBMs and those infiltrating *IDH*-mutant tumors. GBM-infiltrating T cells upregulated multiple genes indicative of a stress-associated, immunoregulatory profile (**Figure 3E**). For example, *CCL20* was elevated in GBM T cells; this chemokine attracts Tregs and could promote an immunosuppressive milieu [27]. *CHI3L1* (encoding YKL-40) was also higher in GBM T cells, consistent with its known association with mesenchymal transition and invasiveness in glioma [28]. GBM T cells showed increased *ISG15* expression, reflecting active type I interferon signaling [29]. By contrast, T cells in *IDH*-mutant gliomas displayed a different phenotype: they had higher expression of *CLDN5*, a tight-junction protein of the blood–brain barrier [30], as well as *RGS5*(involved in vascular homeostasis) and *CAVIN2*, all of which point to a vascular stabilization or peri-vascular localization phenotype [31].

We also compared T cell gene expression between primary and recurrent GBMs (**Figure 3F**). Interestingly, T cells from primary GBMs showed higher *KRT81* expression (a cytoskeletal protein [32]), whereas T cells from recurrent GBMs upregulated *PARD6G* (involved in cell polarity and migration [33]).

Analysis of T cell subset proportions revealed that CD8⁺ T cells were the dominant T cell type in all glioma subtypes, constituting ~40–45% of T cells in astrocytomas, oligodendrogliomas, and GBMs alike (**Figures 3G & H**). GBMs, however, had a higher fraction of proliferative (cycling) T cells than *IDH*-mutant tumors, indicating that a subset of lymphocytes is actively expanding within the tumor. Canonical checkpoint genes (*PDCD1*, *HAVCR2*, *LAG3*) did not differ significantly between GBM and *IDH*-mutant T cells, including within CD8⁺ cells, indicating a stress-associated, immunoregulatory profile rather than classical checkpoint-defined exhaustion, which can coexist with proliferation under chronic antigen exposure. For NK cells, both CD56^bright^ (immune-regulatory) and CD56^dim^ (cytotoxic) NK subsets were more prevalent in GBMs than *IDH*-mutant tumors (**Figure 3D**). This aligns with emerging evidence that NK cells, although present in GBM, may be functionally impaired or excluded in *IDH*-mutant settings [34]. Notably, we did not find significant differences in overall T cell infiltration between *IDH*-mutant astrocytomas vs. oligodendrogliomas (**Figure S5A**). Consistent with prior reports, *MGMT* promoter methylation status was not associated with significant differences in T-cell, NK-cell, or myeloid cell proportions in our cohort, suggesting that *IDH*-mutation status rather than *MGMT* status is the primary determinant of immune infiltration in adult-type diffuse gliomas.Finally, we observed that the GBM-infiltrating Treg subset expressed high levels of *CTLA4* (**Figure S5B**), consistent with an immunosuppressive Treg phenotype in the GBM TME [35].

## Myeloid cell heterogeneity and molecular features across glioma subtypes

Myeloid cells (microglia and macrophages) regulate immune responses and tumor progression in gliomas. We identified three major myeloid subsets across all samples: tissue-resident microglia, infiltrating macrophages, and a proliferative myeloid population containing cycling cells of both microglial and macrophage origin. These subsets were defined by canonical markers and distinct molecular signatures (**Figure 4A & B**),

Analysis revealed substantial inter-subtype differences in the myeloid compartment. Total myeloid cell abundance was significantly higher in GBMs than in *IDH*-mutant astrocytomas or oligodendrogliomas (**Figure 4C**), consistent with the notion that more aggressive gliomas are associated with greater myeloid infiltration [36]. Differential gene expression analysis further revealed that myeloid cells in GBMs, compared with *IDH*-mutant tumors, have distinct activation states (**Figure 4D**). Notably, GBM-associated myeloid cells upregulated genes like *TSPO*, *TIMP1*, *PVT1*, and *LTF*. *TSPO* (Translocator Protein) is linked to microglial activation and pro-inflammatory cytokine production, fostering a tumor-supportive milieu [37]. *TIMP1* (Tissue Inhibitor of Metalloproteinases-1) was elevated in GBM myeloid cells; *TIMP1* can remodel the extracellular matrix, promote angiogenesis, and recruit tumor-associated macrophages [38]. High *TIMP1* levels have been correlated with poor outcomes in GBM [39]. The long non-coding RNA PVT1 was also upregulated in GBM myeloid cells; PVT1 has been shown to drive macrophage polarization toward an immunosuppressive, pro-tumoral M2-like state [40]. Similarly, LTF (lactotransferrin) was higher in GBM myeloid cells, suggesting altered iron metabolism and immunoregulation in the GBM microenvironment [39].

By examining individual myeloid subpopulations, we show that all three subsets (microglia, macrophages, and proliferative myeloid cells) were significantly more abundant in GBMs than in *IDH*-mutant tumors (**Figure 4E**). Microglia, the resident CNS myeloid cells, were especially prominent in GBMs. In the GBM TME, microglia are known to secrete immunosuppressive cytokines (TGF-β, IL-10) and pro-angiogenic factors (VEGF) [39, 41], supporting tumor growth and suppressing immune responses. Infiltrating bone marrow–derived macrophages were also enriched in GBMs and contribute to extracellular matrix (ECM) remodeling and angiogenesis. The proliferative myeloid subset, while a smaller fraction, was specifically elevated in GBM; this population has been associated with myeloid-derived suppressor cell (MDSC)-like activity, capable of dampening T cell responses via production of reactive oxygen species and arginase [42].

Stratification by clinical features identified an increased myeloid infiltration in GBM in both primary and recurrent tumors, with no significant difference between the groups [43]. Similarly, *IDH*-mutant astrocytomas and oligodendrogliomas both had low myeloid content, and *MGMT* promoter status in GBM did not appreciably affect myeloid cell proportions (**Figure 4E**). Thus, consistent with our T cell findings, *IDH*-mutation status emerges as the key determinant of myeloid cell abundance and phenotype in diffuse gliomas.

**State-resolved pathway enrichment reveals distinct metabolic and microenvironment-interacting programs in GBM**

Given that ALPCs and hypoxia-malignant cells are disproportionately enriched in GBMs (**Figure 2C**), we asked whether transcriptional differences within these states between GBM and *IDH*-mutant tumors could help explain the microenvironmental distinctions observed above. We therefore performed differential expression analyses comparing GBM with *IDH*-mutant tumors under ALPC and hypoxia conditions (**Table S4**). We applied Gene Ontology (GO) over-representation analysis to genes upregulated in GBM in each state to identify enriched biological processes (**Figure 5 and Table S5**).

GBM cells in the ALPC state showed striking enrichment of oxidative phosphorylation (OXPHOS), aerobic respiration, and mitochondrial gene regulation pathways (**Figure 5A, B**). The top enriched GO terms included oxidative phosphorylation (74 genes, adjusted p = 2.7×10⁻²⁹), aerobic respiration (88 genes, adjusted p = 1.8×10⁻²⁸), cellular respiration (93 genes, adjusted p = 3.2×10⁻²³), and mitochondrial translation (67 genes, adjusted p = 1.1×10⁻²²), collectively indicating heightened mitochondrial metabolic activity in GBM ALPCs compared to their *IDH*-mutant counterparts. This enrichment of OXPHOS-related pathways is consistent with emerging evidence that glioma stem-like cells maintain elevated mitochondrial respiration and ATP production to support self-renewal, proliferation, and therapy resistance, features that distinguish aggressive stem-like populations from more differentiated tumor cells [44, 45]. It suggests that mitochondrial bioenergetic engagement may be relevant to aggressive GBM biology [46].

By contrast, GBM cells under hypoxic conditions were characterized by enrichment in extracellular matrix (ECM) organization, leukocyte chemotaxis, and detoxification pathways (**Figure 5C, D**). The most significantly enriched GO terms included ECM organization (61 genes, adjusted p = 4.1×10⁻⁷), detoxification (35 genes, adjusted p = 6.5×10⁻⁷), leukocyte chemotaxis (46 genes, adjusted p = 1.2×10⁻⁵), and myeloid leukocyte migration (46 genes, adjusted p = 1.2×10⁻⁵). These enrichments suggest that hypoxic GBM malignant cells express programs consistent with extracellular matrix remodeling and leukocyte/myeloid recruitment signaling, which may contribute to the microenvironmental differences observed in GBM [47]. Hypoxia-driven ECM remodeling has been extensively documented as a driver of glioblastoma invasiveness, involving matrix degradation, integrin signaling, and collagen cross-linking that collectively create permissive corridors for tumor cell migration [48]. Concurrently, the enrichment of chemotaxis and immune-related pathways aligns with established roles of hypoxia in recruiting myeloid-derived suppressor cells (MDSCs), tumor-associated macrophages (TAMs), and regulatory T cells (Tregs) to hypoxic niches, where they contribute to an immunosuppressive milieu that shields tumor cells from immune surveillance [11]. ALPCs exhibit metabolic programs that support stemness and proliferation, while hypoxic cells orchestrate microenvironmental remodeling and immune modulation, collectively contributing to the aggressive, therapy-resistant phenotype of glioblastoma [49].

## Malignant–immune interaction networks reveal state-specific immunoregulatory interfaces

To determine whether expansion of the ALPC and hypoxia states in GBM is accompanied by differences in inferred immune communication, we modeled intercellular signaling using CellChat on composition-matched datasets (90,100 cells per disease group; nboot=100), with communication probabilities computed independently of cluster size (population.size = FALSE). Across independent downsampling seeds, aggregate interaction-weight matrices were near-identical (Pearson r ≥ 0.995) and top-ranked pathways concordant (top-20 Jaccard = 1.00 in both tumor types; **Table S6**), confirming stability under resampling. Here 'information flow' denotes the aggregate signaling strength across a pathway's significant ligand–receptor pairs, and 'communication probability' the inferred strength of a single pair.

Both ALPC and hypoxia states were prominent signaling hubs connecting to immune compartments in both tumor types (**Figure 6A–D**). To compare interfaces on a patient-resolved basis, we ran CellChat independently per tumor and compared malignant-state→myeloid interaction weights with the tumor as the unit of replication (two-sided Wilcoxon rank-sum test; n = 19 GBM, 10 *IDH*-mutant with ≥50 cells at both endpoints). All were higher in GBM: ALPC→myeloid (median 1.06 vs 0.68; Hodges–Lehmann difference 0.34, 95% confidence interval (CI) 0.15–0.53; Benjamini–Hochberg-adjusted p = 0.010; common-language effect size 0.82), hypoxia→myeloid (0.95 vs 0.68; 0.28, 0.05–0.45; adjusted p = 0.016; 0.77), and the summed interface (1.98 vs 1.36; 0.61, 0.23–0.96; p = 0.006; 0.81; **Figure 6E**, **Table S7A**). These differences persisted when each cell group was capped at 200 cells per tumor (**Table S7A**), excluding an artifact of GBM's larger myeloid compartment. *IDH*-mutant tumors contained too few intratumoral T or NK cells to model per patient (0–1 tumors with ≥50 lymphocytes), so lymphoid edges are reported descriptively.

To test whether these differences reflected genuine expression rather than properties of the interaction model, we compared the underlying ligand and receptor genes by per-patient pseudobulk (limma-voom; malignant states as ligand source, myeloid cells as receptor source). Ligand programs were coordinately elevated in GBM in both states (gene-set test p = 5.9 × 10⁻⁵ ALPC, 1.5 × 10⁻⁶ hypoxia), led by SPP1 (log₂ fold-change ≈ 3.7–4.3; adjusted p < 10⁻⁴), MDK, ANXA1, CD99 and laminins; the cognate myeloid receptors were also elevated, more modestly (p = 2.0 × 10⁻⁴), including CD44, CD74, TYROBP, LRP1 and integrins (ITGB1/2, ITGA4, ITGAV). The greater myeloid interaction weights in GBM are thus corroborated by direct, patient-level differential expression of the underlying genes (**Table S7B**).

Decomposing information flow, ALPC and hypoxia shared a core of MIF-, antigen-presentation– and myeloid-associated (APP/APOE) modules; GBM networks additionally featured ECM/adhesion (FN1/laminin/collagen, SPP1) and immunoregulatory (TGFB, NECTIN) families (**Figure 6F–G**) and, at the ligand–receptor level, NECTIN2–TIGIT, TGFB2–TGFBR, PROS1–AXL/MERTK and CD44/integrin (SPP1–CD44) interactions, whereas *IDH*-mutant networks were dominated by APP/APOE–TREM2/TYROBP, MIF–CD74 and antigen-presentation (HLA–CD8/CD4) interactions (**Figure S6A–D**; interactions shown pass CellChat's within-network significance threshold). These decompositions describe network composition and are not statistical tests of between-group differences.

## Spatial dynamics of GBM microenvironment

To provide tissue-level spatial context for the malignant and immune transcriptional programs identified by scRNA-seq, we analyzed spatial transcriptomics data from ten glioblastoma (GBM) samples. We used SPOTlight deconvolution to estimate the relative contributions of broad cell-type classes within each spatial location [50], including malignant cells, glial cells (astrocytes, oligodendrocytes, and oligodendrocyte progenitor cells), immune cells (myeloid and T cells), neuronal cells, and endothelial/ vascular and leptomeningeal cells (VLMC) populations.

Across tumors, we observed pronounced regional heterogeneity in cell-type composition, with marked spatial variation in the relative abundance of malignant, glial, immune, and vascular-associated compartments (**Figure 7A**). Large contiguous regions were frequently dominated by malignant or glial lineage signals, whereas immune and endothelial/VLMC-associated signals were spatially restricted and localized to discrete areas (**Figure 7B**). Regions enriched for T cell or endothelial signatures were not uniformly distributed across tumor sections, consistent with heterogeneous immune infiltration and vascular organization at the tissue scale in GBM. To confirm deconvolution reliability, we reconstructed each spot's expression from its estimated cell-type proportions and the reference profiles and compared it with the observed expression. Per-spot agreement was high across all ten sections (median Pearson r = 0.66–0.95), with correspondingly low root-mean-square error (RMSE) (**Figure S7A and B**), indicating that the estimated cell-type mixtures faithfully reproduce the measured spot expression.

To assess tissue-level spatial co-occurrence (co-location) of major cell-type classes, we calculated pairwise correlations between estimated cell-type proportions across spatial spots (**Figure 7C**). This revealed non-random spatial associations, including positive correlations between malignant cells and glial populations such as oligodendrocyte progenitor–like cells, as well as associations between immune and endothelial/VLMC compartments. These patterns reflect spatial co-enrichment and are consistent with known anatomical features of GBM, such as preferential localization of immune cells in perivascular regions. However, given the resolution and reliance on deconvolution, these associations indicate tissue-scale co-occurrence rather than direct physical contact or functional interaction between individual cell types.

# **Discussion**

A current challenge in adult-type diffuse glioma is to understand how subtype-defining genetics, most notably the *IDH* axis, maps onto reproducible malignant cell states, and how these malignant states align with differences in malignant–immune cell composition across adult-type diffuse gliomas. By integrating single-cell and single-nucleus transcriptomics, we define a shared repertoire of malignant cell states that recur across tumors.In contrast, *IDH*-mutant tumors are dominated by more lineage-restricted, differentiated malignant states, accompanied by a comparatively limited immune infiltrate and a weaker predicted interface between malignant cells and immune compartments.

Prior single-cell studies have shown that glioma cells repeatedly occupy a limited repertoire of transcriptional states [9,13, 23]. Consistent with this, our companion study benchmarked these seven programs against established frameworks and confirmed their reproducibility across independent single-cell cohorts, with ALPCs mapping to astrocyte-like and mesenchymal-like states, the hypoxia state to mesenchymal-like/hypoxia-associated compartments, and the neuro-, oligo- and cycling states to NPC-like, OPC-like and proliferating-progenitor programs [13]. The advance here is integrative rather than a re-identification of states: harmonized quantification of a single state repertoire across the full *IDH*-defined spectrum in one cohort, linkage of state occupancy to modality-controlled immune composition, composition-controlled inference of malignant–immune signaling, and tissue-scale spatial context. We also emphasize that these state distributions are observational associations; whether *IDH*-wildtype status drives ALPC and hypoxia enrichment cell-intrinsically or through a divergent microenvironment cannot be resolved from patient transcriptomes. Separating these possibilities would require lineage tracing or spatially resolved perturbation within intact tumors. Because immune recovery is modality-dependent [16] and less consistent in snRNA-seq, immune-focused analyses were restricted to scRNA-seq, where lymphoid and myeloid subsets were captured with sufficient depth for robust comparison. Because fresh-tissue dissociation recovers lymphocytes more readily than single-nucleus profiling, scRNA-seq may overestimate absolute immune fractions; relative comparisons between subtypes are therefore more reliable than absolute abundances.

Within the malignant compartment, the data most strongly support a shared-state and differential-occupancy view of glioma biology. *IDH*-mutant astrocytomas and oligodendrogliomas preferentially occupy differentiated, lineage-restricted malignant states, consistent with prior observations that *IDH*-mutant gliomas often follow a more differentiated trajectory [9,23]. GBM, conversely, shows higher representation of ALPC, hypoxia, and highly cycling states, aligning with the aggressive clinical behavior of *IDH*-wildtype disease and prior reports linking stem-like and stress-adapted states to malignancy [20–23]. However, we acknowledge that rare or transient cell states and intermediate populations within the broader continuum of glioma heterogeneity may remain uncaptured.

The ALPC state is central to this interpretation because it combines astrocytic lineage features with transcriptional attributes frequently associated with stem-like phenotypes in glioma. We therefore anchored its interpretation using orthogonal evidence. These data are consistent with a malignant phenotype that can be maintained under stem cell-permissive conditions and can undergo differentiation-like responses *in vitro*. However, we could not prospectively purify ALPCs and did not perform *in vivo* tumor initiation, an acknowledged limitation. Our findings also derive from a single cohort and have not been prospectively re-derived in an independent single-cell dataset, which remains an important external validation step. Treatment exposure also differed across the cohort (primary GBMs treatment-naive; most recurrent GBMs chemoradiated) and is a potential confounder of immune differences; however, the principal GBM-versus-*IDH*-mutant differences are already evident among treatment-naive primary GBMs, arguing against treatment as the primary driver, and full treatment history is provided in **Table S1**. Within the *IDH*-mutant group, malignant-state occupancy and immune composition did not differ significantly between astrocytomas and oligodendrogliomas; given the small oligodendroglioma cohort (n=8) these comparisons are underpowered and are not presented as primary analyses. The most rigorous interpretation is therefore that ALPC represents a malignant cell state whose marker program is detectable *in situ* and in patient-derived stem cultures, and whose associated phenotype is compatible with stem-like behavior and differentiation competence *in vitro*, with increased representation in GBM relative to *IDH*-mutant tumors.

State-resolved pathway enrichment provides a mechanistic context for the prominence of ALPC in GBM. Within ALPC, GBM cells are enriched for oxidative phosphorylation, the respiratory chain, and mitochondrial translation pathways,Although enrichment does not establish metabolic dependency, it aligns with evidence that stem-like tumor phenotypes can rely on mitochondrial respiration for growth and state maintenance [44–46]. It also raises the possibility that tumors could evade metabolic pressure through state transitions, motivating functional studies to test whether mitochondrial perturbation alters state occupancy or selectively impacts ALPC-like cells. The hypoxia state defines a complementary axis of specialization. In GBM, hypoxia-state cells are enriched for extracellular matrix organization, leukocyte chemotaxis, and detoxification pathways, consistent with established links between hypoxia, invasion, matrix remodeling, and immune conditioning [48,11]. While pathway enrichment cannot prove causality in vivo, it provides a coherent mechanistic framework that integrates with known GBM biology and predicted malignant–immune interaction networks.

*IDH*-mutant tumors show reduced immune infiltration relative to GBM, consistent with their often sharp tumor borders described histologically [14] and with prior reports of divergent immune landscapes between *IDH*-mutant and *IDH*-wildtype gliomas [25]. Importantly, our lymphoid analyses suggest that the divergence is not purely quantitative. GBM-infiltrating T cells express gene programs consistent with stress and immunoregulatory signaling (including chemokines that may influence regulatory circuits). In contrast, T cells in *IDH*-mutant tumors exhibit expression patterns that are more consistent with vascular-associated and barrier-related contexts. These signatures alone do not establish localization or functional state, such as exhaustion.

The molecular basis of reduced immune infiltration in *IDH*-mutant gliomas has been investigated in experimental settings. Mutant *IDH* produces the oncometabolite (R)-2-hydroxyglutarate (2HG), which has been shown to suppress T-cell immunity through multiple mechanisms. Kohanbash *et al.* [10] demonstrated that *IDH*-mutations suppress STAT1-linked chemokine programs, reducing CD8+ T cell accumulation in glioma models. Bunse *et al.* [51] further showed that 2HG directly suppresses anti-tumor T cell immunity. Together, these findings suggest that reduced expression of key immune-recruiting chemokines (e.g., *CXCL10*, *CCL2*) downstream of 2HG accumulation may contribute to the relative exclusion of lymphocytes observed in our *IDH*-mutant samples.

The myeloid compartment further supports this alignment between tumor entity and immune composition. GBM is enriched for microglia and infiltrating macrophages relative to *IDH*-mutant tumors, consistent with the recognized prominence of myeloid cells in GBM [36,39,41]. Expression of activation and tissue-remodeling-associated genes in GBM-associated myeloid cells (e.g., *TSPO*, *TIMP1*) and regulators linked to immunoregulatory phenotypes (e.g., PVT1) [37–40] supports the idea that GBM immune composition is not merely increased but functionally distinct. While single-cell transcriptomes cannot determine directionality, the convergence of expanded myeloid fractions, state enrichment in ALPC/hypoxia, and state-resolved pathway signatures consistent with remodeling and immune recruitment, collectively motivates a model in which GBM is characterized by coordinated malignant–myeloid coupling.

The communication analyses were structured to assess malignant–immune coupling at the level of defined cell states, while minimizing confounding from cell-number differences through composition matching. Composition matching necessarily reduces rare populations to their shared minimum, so lymphoid-associated inferences are correspondingly less well powered and are treated as exploratory, whereas the well-represented myeloid interface supports the more robust conclusions. We further note that CellChat interaction weights are relative, model-inferred quantities derived from mean ligand and receptor expression rather than measured signaling rates; accordingly, between-group differences in these weights should be read as differences in the expression of interacting ligand–receptor genes propagated through the model, and their absolute values do not carry an independent biological unit. To address this, we compared the malignant-state to myeloid interface between tumors using the patient as the unit of replication and independently corroborated the difference by direct pseudobulk differential expression of the underlying ligand–receptor genes.

Beyond this shared backbone, GBM-associated networks also predict additional immunoregulatory and adhesion/matrix-related signaling families, consistent with established roles for TGFβ-mediated regulation and inhibitory receptor pathways in glioma [35,39] and with the expanded and activated myeloid populations observed in GBM. In contrast, *IDH*-mutant networks were dominated by modules aligned with microglial signaling and baseline antigen presentation. These interactions are inferred from transcriptional co-expression and do not demonstrate protein activity, spatial proximity, or downstream signaling.

Spatial transcriptomics provides a complementary tissue-scale view that supports the regional organization of malignant and immune signals in GBM [52]. At Visium resolution, each spot contains mixtures of neighboring cells, and deconvolution-derived co-occurrence cannot be interpreted as direct cell–cell contact. Nonetheless, the spatial compartmentalization of immune and vascular-associated signals is consistent with a long-standing view of GBM as a regionally structured disease in which immune cells are not uniformly distributed. In particular, spatially resolved regional differential expression and niche-level molecular characterization, for example of perivascular and perinecrotic compartments, represent important extensions that will require higher-resolution, single-cell or near-single-cell spatial platforms to attribute region-defining programs to specific malignant and stromal populations with confidence.

In summary, our data support a state-resolved model in which the *IDH* axis is associated with reproducible differences in malignant cell-state occupancy and malignant–immune composition, with GBM distinguished by expansion of ALPC and hypoxia states that exhibit state-specific functional programs and greater predicted malignant–myeloid interfaces. At the level of specific candidate therapeutic targets, several observations in this study generate testable hypotheses in the context of existing agents or clinical trial frameworks. First, the enrichment of oxidative phosphorylation and mitochondrial respiration programs within GBM ALPC nominates mitochondrial function as a candidate state-specific dependency. Second, the enrichment of NECTIN2-TIGIT interactions in predicted GBM malignant-immune networks is clinically actionable in the context of the expanding TIGIT-directed immunotherapy landscape. Third, SPP1 expression, inferred here as a prominent malignant and myeloid-derived signal engaging CD44 and integrin receptors, is a candidate prognostic biomarker consistent with prior reports of elevated SPP1 associating with poor outcomes in GBM. Finally, the prominence of TGF-β-associated predicted signaling in GBM networks aligns with established roles of TGF-β in glioma immune suppression and has motivated prior clinical trials of TGF-β pathway inhibitors in GBM (e.g., galunisertib). State-resolved analyses of TGF-β ligand expression within ALPC and hypoxia states could help refine patient selection criteria for such approaches. All of these represent data-informed hypotheses, and prospective experimental and clinical validation will be essential before any clinical translation.

# **Methods**

**Patient cohort and sample processing**

The study comprised 69 adult-type diffuse glioma tumor specimens: 44 glioblastomas (GBMs), 17 astrocytomas, and 8 oligodendrogliomas, spanning World Health Organization grades 2–4. Written informed consent was obtained before surgery, and the study was approved by the Bellberry Human Research Ethics Committee (HREC 2019-08-682). Diagnoses and molecular classifications were confirmed by histopathological and molecular assessment. Fresh specimens were profiled by single-cell RNA sequencing (scRNA-seq; 44 specimens, 563,798 cells), archived frozen specimens by single-nucleus RNA sequencing (snRNA-seq; 22 specimens, 75,132 nuclei), and 10 GBM specimens by 10× Genomics Visium spatial transcriptomics. scRNA-seq and snRNA-seq 3′ gene-expression libraries were generated using the Chromium Next GEM Single Cell 3′ platform, sequenced on an Illumina NovaSeq 6000, and processed using Cell Ranger.

**Data processing, integration, and malignant-state classification**

Quality control included removal of ambient RNA, low-quality barcodes, cells with more than 15% mitochondrial reads, and predicted doublets. scRNA-seq and snRNA-seq data were integrated using Harmony, clustered, and annotated into 15 major cell types using reference-based prediction, canonical lineage markers, and manual refinement. Because immune-cell recovery differed between modalities, analyses of immune-cell composition, immune transcriptional phenotypes, and malignant–immune interactions were restricted to scRNA-seq; snRNA-seq was used to extend coverage of the malignant compartment. Malignant cells were classified into seven transcriptional states using non-negative matrix factorization based on a previously described framework [13]. A rank of seven was selected from repeated analyses across ranks 2–25 using reconstruction error and clustering stability.

**Functional characterization of ALPCs**

ALPC-associated markers were assessed by immunofluorescence in patient tumor sections and patient-derived glioma cultures. Four independent cultures maintained under serum-free, stem cell-permissive conditions were evaluated using marker immunofluorescence, neurosphere formation, and a 25-day BMP4 differentiation assay. Differentiation-associated expression changes were quantified by reverse-transcription quantitative PCR relative to RPL13A.

**Differential expression, pathway, and interaction analyses**

Tumor-level pseudobulk profiles were generated using Libra and analyzed using edgeR negative-binomial likelihood-ratio models. Genes with log₂ fold change > 0.5 and Benjamini–Hochberg-adjusted *P* < 0.05 were considered upregulated (a log2 fold-change threshold of 0.5 was chosen to capture coordinated, moderate-magnitude transcriptional programs at pseudobulk, tumor-level resolution, where enrichment reflects pathway-level coherence rather than a small number of large-fold genes) and subjected to Gene Ontology enrichment analysis using clusterProfiler. CellChat v2 was applied to scRNA-seq data after composition-matched downsampling to 90,100 cells in each disease group. Reproducibility was evaluated across three independent downsampling iterations. Communication probabilities were calculated using the trimean estimator with population.size = FALSE. For details on the number of downsampled cells per cell type and the statistical tests performed, see the Supplementary Information. Visium data were processed using Space Ranger and SCTransform, and spot composition was estimated using SPOTlight with annotated snRNA-seq data as the reference [50].

**Statistics**

Tumor-level proportions, rather than individual cells, were treated as independent observations. Comparisons between independent tumor groups used two-sided Wilcoxon rank-sum tests. Statistical tests were two-sided unless otherwise specified, and *P* < 0.05 was considered statistically significant. Detailed Materials and Methods can be found in the Supplementary Information.

**Statements**

**Ethics Statement:** This study was approved by the Bellberry Human Research Ethics Committee (HREC 2019-08-682). Written informed consent was obtained from all participants before surgical resection and sample collection.

**Funding:** This research was supported by a grant from the Charlie Teo Foundation (grant number: PR 10392). Data generation and analysis were partly supported by funding from National Health and Medical Research Council (NHMRC) investigator grants (2009982 and 2034556).

**Conflict of interest:** The authors declare no competing interests.

**Author Contributions:** A.Z., E.H., M.S., and J.E.P. conceived and designed the study. A.Z., M.P., GS., and CE. performed the experiments. R.Z., M.S., J.Y., W.M., and A.M. contributed to experimental design and provided feedback throughout the study. C.T. and J.Y. identified the patient cohort and provided clinical data. A.Z., E.H., W.M., G.S., and K.K. analyzed the data. A.Z., E.H., W.M., and J.E.P. wrote the manuscript with input from M.S. and J.Y. All authors have read and approved the final manuscript.

**Data availability:** Processed single-cell data are additionally accessible through the CZ CELLxGENE Discover portal (https://cellxgene.cziscience.com/collections/384e373e-4db1-4605-812f-d9da7cfd246f; dataset 8f3cb9c4-38d5-4b9c-b309-bcccd76d0075). All code used for data processing and analysis is available at github.com/powellgenomicslab/brain_cancer_paper. Any additional information required to reanalyze the data reported in this paper is available from the lead contact upon reasonable request (j.powell@garvan.org.au).

# **Acknowledgement:** We are deeply grateful to the patients who generously donated their tumor tissue, and to their families for their support. We acknowledge the Charlie Teo Foundation for its funding and support. We also thank the operating room staff at the Prince of Wales Private Hospital for their invaluable assistance in obtaining the tumor sample in a timely manner. Special thanks go to the Garvan-Weizmann Center for Cellular Genomics, including Dr Naushad Moti, for their technical expertise in sample processing and flow cytometry, and Dr Catia Moutinho for her guidance on single-cell capture and library preparation.

# **References:**

1. Louis DN, Perry A, Wesseling P, et al. The 2021 WHO classification of tumors of the central nervous system: a summary. *Neuro Oncol.* 2021;23(8):1231-1251.
2. Sipos D, Raposa BL, Freihat O, et al. Glioblastoma: clinical presentation, multidisciplinary management, and long-term outcomes. *Cancers (Basel).* 2025;17(1):146.
3. Nicholson JG, Fine HA. Diffuse glioma heterogeneity and its therapeutic implications. *Cancer Discov.* 2021;11(3):575-590.
4. Richard Q, Laurenge A, Mallat M, Sanson M, Castro-Vega LJ. New insights into the immune TME of adult-type diffuse gliomas. *Curr Opin Neurol.* 2022;35(6):794-802.
5. Patel AP, Tirosh I, Trombetta JJ, et al. Single-cell RNA-seq highlights intratumoral heterogeneity in primary glioblastoma. *Science.* 2014;344(6190):1396-1401.
6. Neftel C, Laffy J, Filbin MG, et al. An integrative model of cellular states, plasticity, and genetics for glioblastoma. *Cell.* 2019;178(4):835-849.e21.
7. Venteicher AS, Tirosh I, Hebert C, et al. Decoupling genetics, lineages, and microenvironment in IDH-mutant gliomas by single-cell RNA-seq. *Science.* 2017;355(6332):eaai8478.
8. Tirosh I, Venteicher AS, Hebert C, et al. Single-cell RNA-seq supports a developmental hierarchy in human oligodendroglioma. *Nature.* 2016;539(7628):309-313.
9. Varn FS, Johnson KC, Martinek J, et al. Glioma progression is shaped by genetic evolution and microenvironment interactions. *Cell.* 2022;185(12):2184-2199.e16.
10. Kohanbash G, Carrera DA, Shrivastav S, et al. Isocitrate dehydrogenase mutations suppress STAT1 and CD8+ T cell accumulation in gliomas. *J Clin Invest.* 2017;127(4):1425-1437.
11. Haley MJ, Bere L, Minshull J, et al. Hypoxia coordinates the spatial landscape of myeloid cells within glioblastoma to affect survival. *Sci Adv.* 2024;10(20):eadj3301.
12. Liu M, Ji Z, Jain V, et al. Spatial transcriptomics reveals segregation of tumor cell states in glioblastoma and marked immunosuppression within the perinecrotic niche. *Acta Neuropathol Commun.* 2024;12(1):64.
13. Muskovic W, Zaman A, Pandya M, et al. Adult-type diffuse gliomas share recurring cell states driven by a common astrocyte-like glioma stem cell population. Preprint. Posted online June 13, 2024. bioRxiv 2024.06.13.598923.
14. Saito R, Motomura K, Ohka F, et al. SURG-23. Establishment of an appropriate glioma surgery system considering molecular and functional boundaries [abstract]. *Neuro Oncol.* 2024;26(suppl 8):viii279.
15. Dadario NB, Boyett DM, Teasley DE, et al. Unveiling the inflammatory landscape of recurrent glioblastoma through histological-based assessments. *Cancers (Basel).* 2024;16(19):3283.
16. Denisenko E, Guo BB, Jones M, et al. Systematic assessment of tissue dissociation and storage biases in single-cell and single-nucleus RNA-seq workflows. *Genome Biol.* 2020;21(1):130.
17. Carén H, Stricker SH, Bulstrode H, et al. Glioblastoma stem cells respond to differentiation cues but fail to undergo commitment and terminal cell-cycle arrest. *Stem Cell Reports.* 2015;5(5):829-842.
18. Verploegh ISC, Conidi A, Brouwer RWW, et al. Comparative single-cell RNA-sequencing profiling of BMP4-treated primary glioma cultures reveals therapeutic markers. *Neuro Oncol.* 2022;24(12):2133-2145.
19. Huang Q, Zhang QB, Dong J, et al. Glioma stem cells are more aggressive in recurrent tumors with malignant progression than in the primary tumor, and both can be maintained long-term in vitro. *BMC Cancer.* 2008;8:304.
20. Couturier CP, Nadaf J, Li Z, et al. Glioblastoma scRNA-seq shows treatment-induced, immune-dependent increase in mesenchymal cancer cells and structural variants in distal neural stem cells. *Neuro Oncol.* 2022;24(9):1494-1508.
21. Park JH, Lee HK. Current understanding of hypoxia in glioblastoma multiforme and its response to immunotherapy. *Cancers (Basel).* 2022;14(5):1176.
22. Gobin M, Nazarov PV, Warta R, et al. A DNA repair and cell-cycle gene expression signature in primary and recurrent glioblastoma: prognostic value and clinical implications. *Cancer Res.* 2019;79(6):1226-1238.
23. Wei Y, Li G, Feng J, et al. Stalled oligodendrocyte differentiation in IDH-mutant gliomas. *Genome Med.* 2023;15(1):24.
24. Zhang J, Feng Y, Li G, et al. Distinct aneuploid evolution of astrocytoma and glioblastoma during recurrence. *NPJ Precis Oncol.* 2023;7(1):97.
25. Kaminska B, Ochocka N, Segit P. Single-cell omics in dissecting immune microenvironment of malignant gliomas: challenges and perspectives. *Cells.* 2021;10(9):2264.
26. Wang F, Cathcart SJ, DiMaio DJ, et al. Comparison of tumor immune environment between newly diagnosed and recurrent glioblastoma including matched patients. *J Neurooncol.* 2022;159(1):163-175.
27. Manini I, Caponnetto F, Dalla E, et al. Heterogeneity matters: different regions of glioblastoma are characterized by distinctive tumor-supporting pathways. *Cancers (Basel).* 2020;12(10):2960.
28. Wurm J, Behringer SP, Ravi VM, et al. Astrogliosis releases pro-oncogenic chitinase 3-like 1 causing MAPK signaling in glioblastoma. *Cancers (Basel).* 2019;11(10):1437.
29. Li C, Wang J, Zhang H, et al. Interferon-stimulated gene 15 (ISG15) is a trigger for tumorigenesis and metastasis of hepatocellular carcinoma. *Oncotarget.* 2014;5(18):8429-8441.
30. Hashimoto Y, Greene C, Munnich A, Campbell M. The CLDN5 gene at the blood-brain barrier in health and disease. *Fluids Barriers CNS.* 2023;20(1):22.
31. Silini A, Ghilardi C, Figini S, et al. Regulator of G-protein signaling 5 (RGS5) protein: a novel marker of cancer vasculature elicited and sustained by the tumor’s proangiogenic microenvironment. *Cell Mol Life Sci.* 2012;69(7):1167-1178.
32. Vaitkienė P, Skiriutė D, Mueller W. DNA methylation and gene expression of COX7A1, SPINT1 and KRT81 in glioblastoma and brain tissue. *Biologija.* 2012;58(2):71-78.
33. Calabrese G, Bennett BJ, Orozco L, et al. Systems genetic analysis of osteoblast-lineage cells. *PLoS Genet.* 2012;8(12):e1003150.
34. Zhang X, Rao A, Sette P, et al. IDH mutant gliomas escape natural killer cell immune surveillance by downregulation of NKG2D ligand expression. *Neuro Oncol.* 2016;18(10):1402-1412.
35. Khasraw M, Reardon DA, Weller M, Sampson JH. PD-1 inhibitors: do they have a future in the treatment of glioblastoma? *Clin Cancer Res.* 2020;26(20):5287-5296.
36. Liu T, Zhu C, Chen X, et al. Dual role of ARPC1B in regulating the network between tumor-associated macrophages and tumor cells in glioblastoma. *Oncoimmunology.* 2022;11(1):2031499.
37. Ammer LM, Vollmann-Zwerenz A, Ruf V, et al. The role of translocator protein TSPO in hallmarks of glioblastoma. *Cancers (Basel).* 2020;12(10):2973.
38. Vaitkienė P, Urbanaviciute R, Grigas P, Steponaitis G, Tamasauskas A, Skiriutė D. Identification of astrocytoma blood serum protein profile. *Cells.* 2019;9(1):16.
39. Shi J, Huang S. Comparative insight into microglia/macrophages-associated pathways in glioblastoma and Alzheimer’s disease. *Int J Mol Sci.* 2023;25(1):16.
40. Zheng Y, Tian X, Wang T, et al. Long noncoding RNA Pvt1 regulates the immunosuppression activity of granulocytic myeloid-derived suppressor cells in tumor-bearing mice. *Mol Cancer.* 2019;18(1):61.
41. Gao X, Li S, Ding F, et al. A virus-mimicking nucleic acid nanogel reprograms microglia and macrophages for glioblastoma therapy. *Adv Mater.* 2021;33(9):e2006116.
42. Crommentuijn MHW, Schetters STT, Dusoswa SA, Kruijssen LJW, Garcia-Vallejo JJ, van Kooyk Y. Immune involvement of the contralateral hemisphere in a glioblastoma mouse model. *J Immunother Cancer.* 2020;8(1):e000323.
43. Steggerda SM, Bennett MK, Chen J, et al. Inhibition of arginase by CB-1158 blocks myeloid cell-mediated immune suppression in the tumor microenvironment. *J Immunother Cancer.* 2017;5(1):101.
44. Vlashi E, Lagadec C, Vergnes L, et al. Metabolic state of glioma stem cells and nontumorigenic cells. *Proc Natl Acad Sci U S A.* 2011;108(38):16062-16067.
45. Watson DC, Bayik D, Storevik S, et al. GAP43-dependent mitochondria transfer from astrocytes enhances glioblastoma tumorigenicity. *Nat Cancer.* 2023;4(5):648-664.
46. Shang E, Nguyen TTT, Westhoff MA, Karpel-Massler G, Siegelin MD. Targeting cellular respiration as a therapeutic strategy in glioblastoma. *Oncotarget.* 2023;14:419-425.
47. Feldman L. Hypoxia within the glioblastoma tumor microenvironment: a master saboteur of novel treatments. *Front Immunol.* 2024;15:1384249.
48. Monteiro AR, Hill R, Pilkington GJ, Madureira PA. The role of hypoxia in glioblastoma invasion. *Cells.* 2017;6(4):45.
49. Agnihotri S, Zadeh G. Metabolic reprogramming in glioblastoma: the influence of cancer metabolism on epigenetics and unanswered questions. *Neuro Oncol.* 2016;18(2):160-172.
50. Elosua-Bayes M, Nieto P, Mereu E, Gut I, Heyn H. SPOTlight: seeded NMF regression to deconvolute spatial transcriptomics spots with single-cell transcriptomes. *Nucleic Acids Res.* 2021;49(9):e50.
51. Bunse L, Pusch S, Bunse T, et al. Suppression of antitumor T cell immunity by the oncometabolite (R)-2-hydroxyglutarate. *Nat Med.* 2018;24(8):1192-1203.
52. Greenwald AC, Darnell NG, Hoefflin R, et al. Integrative spatial analysis reveals a multi-layered organization of glioblastoma. *Cell.* 2024;187(10):2485-2501.e26.

**Figure legends**

**Figure 1. Single-cell and single-nucleus RNA sequencing analysis of human diffuse gliomas. (A)** Overview of the study cohort comprising 66 patients with *IDH*-mutant and *IDH*-wildtype diffuse gliomas, including primary and recurrent glioblastomas (GBMs). **(B)** Uniform Manifold Approximation and Projection (UMAP) visualization of 638,930 cells derived from integrated scRNA-seq and snRNA-seq datasets, clustered into 15 major cell types based on canonical marker gene expression and validated by copy number variation (CNV) analysis. **(C)** Dot size indicates the fraction of cells expressing each gene, and color intensity represents the average scaled expression level across cells (blue = low, yellow = high). **(D)** Comparison of cellular composition across primary GBMs (pGBM), recurrent GBMs (rGBM), astrocytomas (ASTR), and oligodendrogliomas (ODG), illustrating subtype-specific differences in cell type abundance.

**Figure 2. Cellular state heterogeneity in adult-type diffuse gliomas.** **(A)** Stacked bar chart displaying the relative distribution of malignant cell states within individual tumor samples (GBMs, astrocytomas, and oligodendrogliomas), revealing intertumoral variability and subtype-specific enrichment patterns. **(B)** Dot plot illustrating the expression of representative marker genes across seven transcriptionally defined malignant cell states: astrocyte-like progenitor cells (ALPCs), hypoxia, G1/S proliferative, G2/M proliferative, oligodendroglial (oligo), neuronal–oligodendroglial hybrid (neuro-oligo), and neuronal (neuro). Dot size reflects the proportion of cells expressing each gene, while color intensity indicates the average expression level (blue to yellow). **(C–E)** Grouped bar plots comparing malignant state distributions between tumor subtypes; statistical significance assessed using the Wilcoxon rank-sum test. (**C**), primary and recurrent GBMs (**D**), and lower-grade versus grade 4 *IDH*-mutant astrocytomas (**E**). GBMs show significant enrichment for ALPC, hypoxia, and G2/M proliferative states, whereas *IDH*-mutant tumors predominantly exhibit oligo- and neuro-oligo states. Asterisks indicate statistical significance determined by the Wilcoxon rank-sum test: *p < 0.05, **p < 0.01, ***p < 0.001. All subtype comparisons use tumor-level proportions (one value per tumor) as the unit of replication; p-values reflect across-tumor tests rather than per-cell inference.

**Figure 3. Single-cell transcriptomic profiling reveals distinct T- and NK-cell heterogeneity across adult-type diffuse gliomas. (A)** UMAP visualization of T and NK cells from scRNA-seq data, highlighting transcriptionally defined subpopulations based on canonical marker gene expression. **(B)** Dot plot showing representative marker gene expression across T and NK subpopulations. Dot size indicates the fraction of cells expressing each gene; color intensity represents the average scaled expression level (blue = low, yellow = high). **(C, D)** Box plots comparing the relative abundance (%) of T cells **(C)** and NK cells **(D)** across glioma subtypes. Comparisons include *IDH*-mutant versus *IDH*-wild-type gliomas, astrocytomas versus oligodendrogliomas, primary versus recurrent GBMs, *MGMT*-methylated versus unmethylated GBMs, and lower-grade versus grade 4 *IDH*-mutant astrocytomas. **(E, F)** Volcano plots displaying differentially expressed genes in T cells between GBMs and *IDH*-mutant gliomas **(E)**, and between primary and recurrent GBMs **(F)**. Genes meeting significance thresholds (False Discovery Rate, FDR < 0.05 and |log₂FC| > 1) are highlighted in red (upregulated) and purple (downregulated). **(G, H)** Box plots showing the proportions of T cell subtypes—including CD4⁺, CD8⁺, activated regulatory T cells (Tregs), proliferative lymphocytes, and interferon-stimulated T cells—across *IDH*-mutant gliomas and GBMs **(G)**, and across primary and recurrent GBMs **(H)**. All box plots are based on individual tumor-level quantification. All analyses in this figure are based on scRNA-seq data, as snRNA-seq samples showed very limited recovery of T and NK cells; accordingly, these results characterize transcriptional heterogeneity and relative abundance within the scRNA-seq dataset rather than providing cohort-wide immune quantification. Asterisks indicate statistical significance determined by the Wilcoxon rank-sum test: *p < 0.05, **p < 0.01, ***p < 0.001.

**Figure 4. Heterogeneity and differential abundance of myeloid cell subsets across diffuse glioma subtypes.** **(A)** UMAP visualization of myeloid cells across glioma samples, annotated as microglia, macrophages, and proliferative myeloid cells based on canonical marker gene expression. (**B**) Dot plot showing the expression profiles of representative marker genes for each myeloid subtype. Dot size reflects the proportion of cells expressing each gene; color intensity indicates the average scaled expression (blue = low, yellow = high). (**C**) Box plot comparing the total abundance of myeloid cells across glioma subtypes, stratified by *IDH*-mutation status and histopathological classification. (**D**) Volcano plot displaying differentially expressed genes in myeloid cells between GBMs and *IDH*-mutant gliomas. Genes surpassing differential expression thresholds (FDR < 0.05 and |log₂FC| > 1) are shown in red (upregulated) or purple (downregulated). (**E**) Box plots showing the relative abundance (%) of microglia, macrophages, and proliferative myeloid cells per tumor across stratifications, including *IDH*-mutant versus GBMs, astrocytomas versus oligodendrogliomas, primary versus recurrent GBMs, and *MGMT* methylation status. Box plot values reflect the proportion of each myeloid subtype relative to total immune cells. Asterisks indicate statistical significance determined by the Wilcoxon rank-sum test: *p < 0.05, **p < 0.01, ***p < 0.001.

**Figure 5. Over-Representation Analysis of DEGs using Gene Ontology (GO) database. (A, C)** Dot plot of the top 15 enriched GO terms from upregulated genes of GBM in (**A**) ALPC and (**C**) hypoxia malignant states, ranked by Gene Ratio, then adjusted P values. The significance thresholds used for upregulated genes here were a P value < 0.05 and a log2FC > 0.5. (**B, D**) Category network plot of enriched GO term concepts in (**B**) ALPC and (**D**) hypoxia malignant states annotated with manually curated key genes. Green nodes represent category items, and node size corresponds to gene count. Surrounding nodes are genes associated with that item, colored by log2FC. Overall, GBM cells in the ALPC state show clear enrichment in cellular respiration and mitochondrial gene regulation, whereas GBM cells in the hypoxia state show enrichment in extracellular matrix organization processes and chemotaxis/detoxification.

**Figure 6. CellChat-inferred ALPC- and hypoxia-associated malignant–immune signaling across the *IDH* axis.** **(A–D)** Circle plots summarize outgoing ligand–receptor communication predicted from malignant sender states (ALPC or hypoxia) to immune receiver populations (myeloid, T cells, NK cells) in scRNA-seq cohorts analyzed separately for GBM (**A, C**) and *IDH*-mutant gliomas (**B, D**). Edge thickness denotes the aggregate communication probability, calculated as the sum of interaction weights across ligand-receptor pairs retained as significant (*p* < 0.05; CellChat permutation framework) for each sender-receiver pair, from a single composition-matched run shown for descriptive orientation; these are relative, model-inferred quantities and do not carry an independent biological unit. **(E)** Per-tumor comparison of aggregate malignant-state→myeloid interaction weights between GBM and *IDH*-mutant glioma. CellChat was run independently for each tumor; each point is one tumor, and boxes show the median and interquartile range for ALPC→myeloid, hypoxia→myeloid, and their combined sum (n = 19 GBM, 10 *IDH*-mutant with ≥50 cells at both endpoints). p-values are two-sided Wilcoxon rank-sum tests (Benjamini–Hochberg-adjusted for the two individual edges; unadjusted for the combined interface); full statistics and the independent expression-level corroboration are reported in **Table S7**. Lymphoid edges are not shown as too few *IDH*-mutant tumors contained sufficient T or NK cells for patient-level testing. **(F, G)** Stacked bar plots show the top signaling pathway families contributing to ALPC→immune (**F**) or hypoxia→immune (**G**) communication; the x-axis indicates relative information flow, with bar colors denoting proportional contribution from GBM (red) versus *IDH*-mutant glioma (teal). This decomposition is descriptive and is not a statistical test of between-group differences. Information flow represents the aggregate signaling strength across all significant ligand–receptor pairs for each pathway. Analyses used CellChat v2 on scRNA-seq data with cell-type–balanced downsampling (90,100 cells per disease group) to minimize compositional effects. Communication probabilities were inferred using the trimean estimator of average expression, and significance was assessed by permutation (*nboot* = 100). Inferences reflect transcriptome-based predictions rather than direct protein-level measurements.

**Figure 7. Spatial organization and spot-level co-enrichment of major cell-types in GBM (Visium). (A)** Pie charts summarizing the relative contributions of major cell-type classes across ten GBM sections profiled by Visium. Cell-type classes (malignant, glial, immune [T cells and myeloid], neuronal, endothelial/VLMC) were estimated per Visium spot using SPOTlight deconvolution. **(B)** SPOTlight-derived spatial maps showing the distribution of major cell-type class proportions across each GBM section. Color intensity represents the estimated proportion per spot (scaled 0–1). **(C)** Spot-level co-location (co-occurrence) analysis: correlation matrix summarizing pairwise linear associations between deconvolved cell-type proportions across Visium spots (aggregated across sections). Positive values indicate spot-level co-enrichment and negative values indicate spatial segregation at Visium resolution. These associations reflect tissue-scale co-occurrence within multicellular spots and should not be interpreted as direct cell–cell contact or functional interaction. (n = 10 GBM sections).
